# Reply to Edward, R. Comment on “Fernandez Martin et al. Analgesia for Upper Abdominal Surgery, a Scoping Review of the Current Fascial Plane Block Techniques. *J. Clin. Med.* 2025, *14*, 8632”

**DOI:** 10.3390/jcm15083152

**Published:** 2026-04-21

**Authors:** Maria T. Fernandez Martin, Edward R. Mariano, Luis F. Valdes-Vilches, Servando Lopez Alvarez, Nabil Elkassabany

**Affiliations:** 1 Department of Anaesthesiology, Rio Hortega University Hospital, 47012 Valladolid, Spain; maitefm70@hotmail.com; 2 Department of Anaesthesiology, Perioperative and Pain Medicine, Stanford University School of Medicine, Stanford, CA 94305, USA; 3 Department of Anaesthesiology, Costa del Sol Hospital, 29603 Marbella, Spain; 4 Department of Anaesthesiology, Abente y Lago Hospital, 15006 A Coruña, Spain; 5 Department of Anesthesiology, University of Virginia, 1215 Lee Road, Charlottesville, VA 22903, USA

Dear Editor,

We would like to thank Dr. Reginald Edward for taking interest in our review of the fascial plane blocks used for analgesia after upper abdominal wall surgery [1] and for his insightful comments about the evolution of modern regional analgesia in the era of enhanced recovery. His comments are mainly focused on epidural analgesia and the concern that it has been “*prematurely de-prioritised without adequate exploration of modernised delivery strategies*”. He presents the point of view that fascial plane blocks should be viewed as adjuncts rather than alternatives to epidural analgesia, and he concludes by calling for a re-evaluation of the role of epidural analgesia within the enhanced recovery framework considering new paradigms for epidural dosing and management. We agree that changes in practice should be based on evidence demonstrating better clinical outcomes, whenever available, rather than convenience or fashion. However, the main aim of our scoping review was narrating the evidence for fascial plane blocks used for abdominal surgery, and we had no intention of directly comparing epidural analgesia to fascial plane blocks, although some of the cited studies may have done that. We agree that changing delivery and dosing regimen may result in improving patient mobility. Whether patient outcomes will improve is debatable.

Contemporary perioperative care is increasingly defined by multimodal analgesia, and we agree with Dr. Edward that a thoracic epidural remains the ‘gold standard’ technique for regional analgesia after major open upper abdominal surgery [2] and continues to have important applications when optimally delivered within an experienced system. Epidural analgesia provides reliable somatic and visceral pain control. However, recent meta-analyses show that thoracic epidural analgesia provides only a marginal benefit over intravenous morphine and is no more effective than other regional techniques [3,4]. It is also important to note that there is an increasing number of patients receiving anticoagulant or antiplatelet therapy for whom safer regional alternatives are required [5]. We have listed the limitations of the current published literature in the scoping review and have supported the fact that fascial plane blocks generally address somatic pain and not visceral pain.

We share the concern that institutional practice may be influenced by “operational convenience and risk aversion” [6], contributing to a decline in expertise in thoracic epidural placement. The failure rates reported in the literature vary between 23% and 28% [7], thus emphasising the necessity for enhanced training and workflow. Complementary technologies, including fluoroscopy, epidural waveform analysis and electrical stimulation, have been shown to reduce failure rates to as low as 2% [8]. However, these technologies remain underutilised in routine practice. Most epidurals are still administered using the conventional loss-of-resistance technique in the operating theatre and are typically performed in a hasty manner due to the numerous preoperative tasks that must be completed prior to the commencement of surgery. If the patient requires an effective thoracic epidural, it must be prioritised.

Our position is not that epidural analgesia has been rendered obsolete, but that contemporary perioperative care must also accommodate effective and scalable alternatives for patients in whom epidurals are contraindicated, declined, technically unsuccessful, or impractical to deliver consistently [9]. Rather than viewing regional analgesia as “all or none” (i.e., epidural or nothing), we take a more pragmatic view that all patients having major abdominal surgery should receive a regional technique as part of a multimodal analgesic regimen that is appropriate for their circumstances. And, although the literature on the subject suggests that fascial plane blocks may not reliably treat visceral pain, they have nonetheless been presented as a valid alternative to epidural analgesia for consideration within the context of multimodal analgesia and enhanced recovery [10,11].
